# Enduring strengths and a critical gap, the Ottawa Charter and animal rescue fostering in Aotearoa-New Zealand

**DOI:** 10.1093/heapro/daag078

**Published:** 2026-06-09

**Authors:** Christine Roseveare, Linda Murray, Mary Breheny, Juliana Mansvelt, Marg Wilkie

**Affiliations:** School of Health Sciences, Massey University, P O Box 756, Wellington 6140, New Zealand; School of Health Sciences, Massey University, P O Box 756, Wellington 6140, New Zealand; School of Psychology, Massey University, Private Bag 11 222, Palmerston North 4442, New Zealand; School of People, Environment and Planning, Massey University, Private Bag 11 222, Palmerston North 4442, New Zealand; School of Health Sciences, Massey University, P O Box 756, Wellington 6140, New Zealand

**Keywords:** animals, health promotion, ‘human animal interaction’, fostering, care

## Abstract

According to the Ottawa Charter, both care for others and a supportive environment promote health. Although the Ottawa charter focuses on the human context, animal rescue organizations can be conceptualized as providing supportive environments as part of their work to rehome animals. Animal fosterers also build care relationships with the animals they foster. Therefore, the Ottawa Charter provides a foundation for viewing animal fostering as health promotion. This mixed-methods study analysed animal rescue cat fostering programmes in Aotearoa-New Zealand from the perspective of the Ottawa Charter. Data was collected using a quantitative survey of 40 cat rescue organizations with foster programmes, and semistructured interviews (*n* = 14) with foster programme co-ordinators across Aotearoa-New Zealand. Template analysis was used to apply principles from the Ottawa Charter to the interview data, revealing (i) fostering as care to create health, and (ii) Ottawa charter strategies in action. Key findings were that fostering involved multiple, sometimes challenging aspects of care for others. Some co-ordinators described using strategies aligned with Ottawa Charter action areas to structure their service (strengthening community action, creating supportive environments, developing skills, and reorienting from institutional to community-based services). These strategies supported their ability to care for themselves, and animals. We argue the Ottawa Charter's framing of care for others can be expanded to include human–animal relationships and that cat fostering programmes demonstrate health promotion in action. This case study demonstrates the Ottawa Charter's enduring contribution while highlighting the need for public health to evolve to maintain relevance in our multispecies world.

Contribution to Health Promotion

- Animal fostering can be considered health promoting from a broad, relational perspective, complementing perspectives which focus on individual health outcomes.
- Community cat fostering represents care for others in an everyday setting and promotes health for both people and animals.
- Community cat fostering uses strategies from the Ottawa Charter—strengthening community action, reorienting health services, and creating supportive environments.
- The Ottawa Charter's framing of care for others can expand to encompass human–animal relationships.

## Introduction

From a Health Promotion perspective, our contributions to others matter.

The Ottawa Charter for Health Promotion argues that health is created and lived by people within the settings of their everyday life where they learn, work, play, and love, in part through care for others (World Health Organization 1986). The WHO defines a setting as a place or context in which people engage in daily activities where factors interact to affect health and well-being (Aagaard-Hansen and Bloch 2023). Along with the acknowledgement that health is created and maintained in ‘everyday settings’, the Ottawa Charter recognizes the impact of the broader environment on health (Thomas *et al*. 2025), calling for society to create conditions that allow the attainment of health for all. The five action areas of the Ottawa Charter for health promotion recognize this wider focus. Along with developing personal skills, identified strategies are strengthening community action, creating supportive environments, strengthening public policy, and reorienting health services. The five strategies draw upon a socioecological perspective which recognizes that interactions between individuals and their immediate and wider environment influence health (McLeroy *et al*. 1988, Heard and Bartleet 2025).

Care for others inherently involves relationships. Recently, health scholars have argued for the importance of including relationships with, and responsibilities for animals in our understanding of health promotion (Stephen 2020, Hoy-Gerlach and Townsend 2023, Toohey 2023, de Leeuw *et al*. 2024). Building on the Ottawa Charter's premise that health is ‘created by caring for oneself and others’, Rock *et al*. (2015) contends that this caring relationship should encompass nonhuman others, particularly in the context of relationships with companion animals or pets. In a similar vein, Travers *et al*. (2022) argue that in disaster settings, the importance of relationships between people and companion animals make the health and safety of pets a health promotion concern.

Building on this foundation of existing literature and the Ottawa Charter, our review paper (Roseveare *et al*. 2023) argued that animal fostering can be understood as health promotion. The purpose of the present paper is to investigate the health promotion potential of cat foster programmes within animal rescue organization settings, using an Ottawa Charter lens.

### Companion animal fostering and health promotion

Increasingly, we live in a world of multispecies households where humans share a home with one or more nonhuman animal companions, and think of these animals as members of the family (Companion Animals New Zealand 2020, Applebaum *et al*. 2021, Forrest *et al*. 2023). Aotearoa-New Zealand is a nation of pet owners^1^ with 40% of households having at least one cat, the most common companion animal (Companion Animals New Zealand 2024). Nevertheless, managing and rehoming stray, unwanted, and abandoned animals is a challenging issue for animal rescue organizations. In Aotearoa-New Zealand, animal rescue organizations attempt to find homes for thousands of companion animals every year (Roseveare and Gates 2024).

An increasing number of companion animal rescue organizations (CARO) use community foster programmes, where stray or surrendered animals are placed in temporary foster homes. Volunteer fosterers care for animals at a time of transition until they are ready to be adopted (Canfield *et al*. 2011, Roseveare and Gates 2024) or can be reunited with their original human guardian. Animals may be fostered because they are too young to rehome, would benefit from socialization, for medical or behavioural rehabilitation (Patronek and Crowe 2018, Carver 2020), because of a lack of capacity in an animal shelter, or in response to an emergency event (Kogan *et al*. 2004, Campbell 2021). Community fosterers live, work, and play with foster animals for periods ranging from a few days to several months or longer. Fostering may also involve a longer-term arrangement designed to ensure permanent homes for harder-to-rehome animals.

Companion animal fostering programmes play an important role in animal health by increasing the capacity of animal shelters to accept stray or surrendered animals (Horecka and Neal 2022, Reese *et al*. 2022), reducing stress on animals (McDonald *et al*. 2022), reducing the risk of euthanasia in the shelter system (Kerr *et al*. 2018, Patronek and Crowe 2018), and increasing the likelihood of adoption (Daily 2021, Reese *et al*. 2022). Fostering animals in a home environment can reduce the animal's exposure to infectious disease, allow sick or injured animals time to heal, and provide opportunities for socialization (Reese *et al*. 2022). Socialization refers to the process of introducing an animal to a variety of different experiences, environments, people, and other animals (SPCA New Zealand n.d). Socialization increases the chance of successful rehoming with a human family. Community foster programmes play a significant role in Aotearoa-New Zealand cat rescue, with 80% of CARO who care for cats having a foster programme. Fifty-nine percent of cats currently under the care of cat CARO are located with fosterers, and the availability of fosterers is identified as the most important factor affecting their ability to accept animals (Roseveare and Gates 2025).

Fostering may have benefits for human fosterers as well as the animals they care for, although the research is limited. Studies where an older person cared for a foster animal as an intervention to improve health found modest improvements in quality of life (Ortmeyer and Robey 2019), and reduced loneliness (Sanderson *et al*. 2023). Regular fosterers value the social ties, sense of purpose and community contribution, and connection with animals they gain through fostering (DeWitt 2020, Phillips and Gunter 2024, Roseveare *et al*. 2025). However fostering relationships are not without challenges, including dealing with difficult emotions, such as the grief of relinquishment or death of a fostered animal (Barrett and Patlamazoglou 2018, Powell *et al*. 2024, Roseveare *et al*. 2025).

Intervention studies treat the fostering relationship as an epidemiological exposure, that leads to specific individual beneficial health outcomes. For example, Powell *et al*. (2024) surveyed fosterers before and after fostering an animal and concluded that fostering did not promote mental wellbeing because psychometric mood measures did not improve. Yet despite this finding, fosterers in their study reported that their foster animal provided love and affection, and the majority intended to foster again. The findings of this study highlight different perspectives regarding health promotion. From an epidemiological perspective, positive changes in specific quantitative indicators of health before and after a fostering event would make it health promoting.

However, a broader health promotion perspective allows for human animal relationships to be more complex—both simultaneously supportive and challenging, and understood within the context of a relational, socioecological framework (Toohey 2023). Health understood as a resource for everyday life rather than an endpoint, can be enhanced through relationships of care even when these relationships involve difficult experiences. From a broad, eudaimonic definition of wellbeing (which incorporates purpose in life, positive relationships, maturity, and personal growth) (Ryff 2017) challenging experiences of providing care still contribute to a sense of meaning, wellbeing, and the living of a full life.

Considering whether animal fostering is health promoting from an Ottawa Charter perspective enables at least three lines of enquiry that might otherwise be overlooked. First, it expands what ‘health promoting’ means to include community connection, sense of purpose, and contribution to others—including relationships with other species. Second, it acknowledges that relationships can be simultaneously challenging ‘and’ health promoting, rather than requiring challenges to be eliminated for fostering to be beneficial. Finally, it enables examination of how rescue organizations may function as health promotion settings that enable meaningful participation in caring relationships. By identifying alignments between fostering and health promotion frameworks we can encourage a reconceptualization of those frameworks to encompass human–animal relationships. The overall objectives of this study were to (i) identify why rescue organizations use foster programmes and (ii) analyse cat fostering as a case study through the lens of the Ottawa Charter for health promotion.

### Cat fostering in the Aotearoa-New Zealand context

In Aotearoa-New Zealand, the Ottawa Charter sits alongside Te Tiriti O Waitangi as forming the foundation of health promotion (Health Promotion Forum of New Zealand 2002). Alongside this, an Indigenous Māori model of health, ‘Te Whare Tapa Whā’ ‘the four sided house’ (Durie 1994), emphasizes the broad and interdependent nature of health which aligns with the Ottawa Charter. These principles are further developed in the Geneva Charter for Wellbeing (World Health Organization 1986, 2021). Both *Te Whare Tapa Whā* and the Geneva Charter acknowledge Indigenous perspectives (Fraser-Celin and Rock 2021), conceptualizing health as integrating physical, mental, spiritual and social well-being.

Māori are the Indigenous people of Aotearoa-New Zealand. Aotearoa-New Zealand health promotion perspectives recognise the value of mātauranga Māori (Māori knowledge or ways of knowing), alongside Western understandings of connections and relationships between humans and animals (Woodhouse *et al*. 2021). Important concepts *are whakapapa* or connection between all things which expresses the relationships that tie Māori to the nonhuman world, including animals (Stewart 2024) and ‘kaitiakitanga’—guardianship or stewardship. The concept of kaitiakitanga justifies both the importance of care for animals as a group and at an individual level. Māori also recognize animals may take on the role of kaitiaki or guardians for humans (Woodhouse *et al*. 2021, Stewart 2024). These perspectives have shaped our understanding as researchers of health promotion, and the importance of more than human relationships.

This study is part of a wider research programme on the health promotion potential of animal fostering for older people in Aotearoa-New Zealand, with a focus on cat fostering for rescue organizations. The aim of the study was to explore feline companion animal fostering through a broad public health, health promotion lens. The research questions were (i) Why do rescue organizations use foster programmes and (ii) What does it mean to bring a public health/health promotion perspective to companion animal fostering in the context of Aotearoa New Zealand.

## Materials and methods

This was a mixed methods study, with data collected using a cross-sectional survey, supplemented by qualitative in-depth interviews.

### Survey

A questionnaire was emailed to all CARO who were registered on the charities database. To identify CARO that were not registered charities, we asked national animal welfare organizations and local rescue networks to identify additional rescue organizations. A Google search was also conducted using keywords such as ‘animal rescue’, ‘dog’, ‘cat’, ‘fostering’, and ‘pet adoption’ to identify organizations not on any other lists. We created a combined list from these sources, excluding organizations that solely provided services for wildlife or marine life. The survey was completed by 106/208 (51%) of the final list of CARO, distributed across the country.

The objectives of the survey were (i) to establish a basic census of animal rescues and fostering organizations in Aotearoa-New Zealand to characterize their number, location, and capacity and (ii) to understand the unique challenges facing each organization in accomplishing their goals. The questionnaire was divided into four main sections. Section Discussion for CAROs with a cat fostering programme, is reported on here. The process for generating the sampling frame, developing and distributing the survey, and analysis of other sections of the survey is available elsewhere (Roseveare and Gates 2024, 2025).

The options offered in the survey were based on concepts and metrics used in animal shelter literature (Stavisky *et al*. 2012, Shelter Animals Count 2023). Survey questions relevant to this paper asked about the kind of fostering programme offered and the perceived advantages and disadvantages of fostering programmes. Interview questions expanded on these questions by asking about the organization overall, why they used foster programmes, and asking specifically about the benefits and challenges for animals, humans, and the organization.

### Interviews

Organizations with a foster programme with at least three fosterers who responded to the survey, were invited to participate in a follow-up interview to discuss their foster programme. Of 18 respondents who expressed initial interest, 14 interviews were completed: twelve were conducted via Zoom and two face to face. At the request of the participants, three interviews included a second person who the participant believed could add useful additional insight. Interviews took from 45 to 60 min. The final group of participants represented a range of rescue organizations from small organizations with only three fosterers to those with 30 or more. All but one were registered charities. All participants had some involvement in, designing or supporting foster care and all but one were female. For simplicity all respondents are referred to as ‘co-ordinators’. The study was reviewed and approved by the Massey University Human Ethics committee (SOB 21/59).

We approached this study from a critical realist perspective. Critical realism aligns with a broad health promotion perspective, as it emphasizes both wider contextual influences and the experience and diverse perspectives of individuals (Maher *et al*. 2025). Critical realists will often seek to draw on or develop existing theory or frameworks, in a specific area (King and Brooks 2017), using a mix of methods to identify patterns and processes (Maxwell 2012). We both drew on and sought to develop health promotion theory through researching foster care in the animal rescue setting, and the wider context it is part of.

From a critical realist perspective, a real world exists independent of our beliefs and constructions of that world. However, our apprehension of the world is shaped by our different experiences and backgrounds (Maxwell 2012). We recognize that our experience, disciplinary background, and perspectives influence our research findings. The research team are multidisciplinary, bringing together expertise in health promotion, social gerontology, and kaupapa Māori perspectives. One researcher is an experienced animal fosterer.

### Survey data

All survey data were imported into R statistical software version 4.2.1 (R Development Core Team, R Foundation for Statistical Computing, Vienna, Austria) for cleaning and analysis. Any duplicate responses from the same organization were removed, and only responses from individuals who completed at least 20% of the survey questions they were eligible to answer were retained for initial analysis. In total, 70 responses were excluded from the initial analysis and 106 were retained. Analyses were restricted to the organizations who responded that they cared for cats (*n* = 64), responded to at least 20% of the survey questions which dealt with cats and fostering (50/64; 78%) and had a fostering programme (40/50; 80%).

### Interviews

Interview transcripts and qualitative data from the free-text comments for survey questions were imported into Quirkos software (Quirkos 2024) and analysed using template analysis (King 2012, King and Brooks 2018). Template analysis is a structured form of thematic analysis where themes identified by a researcher are organized into a codebook or ‘template’ that represents the relationship between different themes. Broad overarching themes encompass lower levels of more specific themes. The initial coding template was created using a subset of data, applied to further data, iteratively reﬁned and then applied to the whole data set. We initially developed codes to represent the organization, delivery and perceived benefits and disadvantages of fostering from the perspective of co-ordinators. In a second cycle of coding (Saldaña 2016), we recognized alignment between the codes and aspects of the Ottawa Charter, so reorganized the template around Ottawa Charter concepts. The template was iteratively refined to incorporate different aspects of the Charter. Both the survey data and the interview data were considered when developing themes. Theme development was led by the interview data, with survey responses considered alongside to provide context and inform the process.

The Ottawa Charter provides three foundations to explore our conceptualization of fostering as health promotion, and our coding framework reflects these. The first is the Charter's focus on care for others as part of what creates health. Here, we considered whether fostering is fundamentally a relationship of care and how it creates health. Second, we consider the settings where fostering activities take place, and wider challenges impacting on these settings. Finally, we looked for examples of Ottawa charter strategies that foster organizations used to support foster care delivery.

## Results

Table 1 presents our analysis of co-ordinator interviews organized according to these three Ottawa Charter components. The table shows our coding template which used Ottawa charter components as level 1 themes, and aspects of these components as level 2.

**Table 1:** Coding template first and second level themes.

Level 1	Level 2
1. Fostering as care to create health	1. It's about outcomes…built on relationships
	2. Animal health and wellbeing
	3. Human health and wellbeing
	4. Caring relationships simultaneously benefit and challenge
	5. Care constrained by a challenging context
2. Ottawa Charter Strategies in action	1. Reorienting the health system
	2. Strengthening community action
	3. Creating supportive environments
	4. Developing individual skills

### Fostering as care to create health

The theme of care was central to organizations’ decisions to use fostering programmes. While influenced by a broad range of factors, at its heart fostering is about care. Care ‘about’ animals—that they should live and find a home, care expressed ‘through’ policies and systems to support rehoming—and care for animals ‘by’ fosterers. These elements of care were expressed across the analytic themes.

#### It's about outcomes…built on relationships

Fostering was described as ‘all about the cats’ and achieving good outcomes for them. Placing animals in foster care was seen as part of a process to achieve the eventual long-term outcome of improved health for animals through rehoming, with the needs of animals being central.

And our goal is to bring them into care, we place them in foster care, and hopefully find a forever home for each and every one of them. (ID 103)

To achieve this outcome, co-ordinators emphasized the importance of socializing animals through the human–cat relationship. Some spoke of the care they took to ‘match’ the cat not just to the best final adoptive home, but with the most appropriate fosterer and environment for both cat and fosterer.

We look at the whole, who’s a good match on paper, and then when they come in, how do, how do we like them? But more importantly, does the cat like them? (ID 9)

The concept of matching was spoken of as a process that could directly impact final outcomes. Foster carers learn about the needs and preferences of the animals. This helped to ensure potential adoptees would know more about the animal before choosing them, enabling matches to permanent homes that would ‘stick’.

So it’s not as if you’ve got a shelter where somebody…like a supermarket somebody rocks up ‘Oh I like the look of that one I’ll have that.’.. we do try very hard to match. (ID 37)

#### Animal health and wellbeing

Rescue organizations used community foster programmes because of the perceived contribution to animal wellbeing.

they get the love and the health, the shelter, all the four or five needs of animals, you know, love, shelter, safety, food, warmth. Yeah. Get those basic needs met and then on top of that they, you know, become part of a family. (ID 86)

Subject to having enough fosterers, foster care programmes increased the capacity of organizations to rehome animals. Foster programmes also allowed organizations with specialized treatment facilities to focus on high needs cases requiring specialist care. The perceived benefits of foster programmes for cats based on survey responses are shown in Table 2. The most frequently identified was more opportunity for enrichment and socialization (93%) followed by less stress for animals being in a foster home (80%).

**Table 2:** Contribution of fostering to animal wellbeing.

Response	Number	%
More opportunity for enrichment and socialization	37	93%
Less stressful for animals to be in a foster home	32	80%
Improves the chances of rehoming/adoption	30	75%
Reduces chance of disease spread	29	73%
Can accept more animals (stops overcrowding or lack of space at the shelter)	25	63%
Denominator	40^a^	–

^a^More than one response allowed.

Our interview findings reinforced the importance of socialization. Positive socialization experiences impact the long-term health and welfare of cats, as well as the likelihood that they will be successfully rehomed (Graham *et al*. 2024). Disadvantages for the animals were rare; potential disadvantages suggested in survey responses were that inexperienced fosterers might fail to pick up on illness, unintentionally hurt animals, or allow animals to escape from the foster home.

#### Human health and wellbeing

Co-ordinators, many who were also fosterers themselves, described the potential benefits of fostering animals for the health of the human fosterer. They described fostering as emotionally rewarding for fosterers, allowing the opportunity to enjoy the company of an animal, providing a sense of contribution without long-term commitment or significant cost: ‘Well you know, it's well documented the feelgood factor with volunteering and helping and doing something in the community for free. So making that contribution’ (ID86).

They noted too, the sense of purpose provided by, and experienced through, fostering and the family connection it could provide:

A lot of our fosters want, what they get out of it is they want to help. And they, you know, this is something they can do in their homes… and get their kids involved. A lot of people like the idea of their kids having to be responsible to help raise these kittens. (ID56)

#### Caring relationships simultaneously benefit and challenge

Co-ordinators described the multilayered experience of fostering, encompassing not only love and care, but also grief and loss. Fostering may be challenging in part because of the temporary nature of fostering

we have some foster homes who have been working with us for years and they cry every time a cat goes. But it’s kind of a nice, it’s a nice, I guess it’s a bittersweet time, because they’re happy. I guess the goal of what, the goal is goodbye, to say goodbye because they get their forever home. (ID 103)

The death of a fostered cat can be a particularly distressing experience but even this was still framed in terms of relationships of care. In the quote that follows the participant highlighted how caring through death can contribute to a meaningful life for both the animal and carer.

And it does hurt you know. Like if I have to take a cat to be put down…I’ll cry all the way there and cry all the way back. But, you know, at least that cat knew some love while we had it. And somebody’s crying for that poor cat. Yeah, so… It is, it’s heartbreaking work but it’s also very, very rewarding. (ID16)

Co-ordinators’ recognized stressful or painful experiences could still be valuable as manifestations of emotional connection. Together the quotes in this section demonstrate that fostering provides rewards (contribution, purpose, family connection) ‘and’ challenges (grief, loss, heartbreak), sometimes at the same time. This complexity was recognized as part of meaningful caring work.

#### Care constrained by a challenging context

Animal rescue fostering exists in a challenging financial context including organizational constraint and cost pressures for households. One of the biggest factors affecting Aotearoa-New Zealand animal rescue organizations is funding ‘we are 100% reliant on donations’. While animal ‘control’ is funded, rescue and rehoming organizations receive no government funding and rely on grants, donations or self-funding to support their work (Roseveare and Gates 2024).

This is what you need to know> The limit varies from time to time, it can be our capacity, it can be the number of fosterers, it can be the amount of money we’ve got, or it can be the ability of the vets to help us because of their capacity…so there’s lots of things. (ID 32)

Co-ordinators also spoke of challenges to families and households that shaped fostering. Most foster organizations provided food, medications, and veterinary checks, meaning fosterers did not need to provide the direct costs of animal care. Most did not cover transport costs, and one co-ordinator commented ‘we’re starting to see a couple [of fosterers] have dropped off because they can’t afford it… they can’t afford the petrol’ (ID 56).

Co-ordinators capacity to care about and for animals is influenced not just through financial constraints but by the availability of fosterers. This in turn is impacted by wider environmental factors such as rental policy.

Each step, there’s kind of a challenge. So getting people interested or reaching people to let them know it’s an opportunity, and then onboarding right now, it’s still a challenge for us…And one of our other challenges with fostering is the rental climate in New Zealand which is quite challenging. (ID 24)

Overall, challenges and constraints include accessing and funding veterinary care, a rental environment unfavourable to pets, costs of transport, lack of funding, and the lack of capacity to respond to the constant flow of homeless animals. The challenging environment alongside capacity limitations meant that cats in need must be turned away: ‘And people just keep messaging or wanting us to take more and more and more. And we’ve got to say no, just we’ve got to make a cut-off point. And that’s really, really hard to do’ (ID82).

Despite the challenging setting, the value placed on animals and the potential for them to give and receive love provides motivation for this caring work.

When you think of, you know, these tiny little things, and they’ve got mucky eyes and they’ve got diarrhoea, and you know, worms and … nobody loves them. They just get a whole new life. …. And that’s the one thing when it was really difficult last year for the cat rescue, I just thought, you know, I said to our group… ‘Do we give up because it’s just too hard?’ And then I just got ‘No, we can’t give up.’ [Pause]. Yeah. So, you know for them they end up being, you know, a really important part of people’s families and they’re so loved. And people say they’re the best cats…and they just adore them. And yeah, and that’s what keeps you going. (ID86)

Care operates at multiple levels, as the co-ordinator describes her sense of commitment to animals and the work of the organization to provide for their care despite constraints. She describes the role she sees her work playing in enabling future valued care relationships between humans and animals. While the ability to practice care is constrained by the wider context, a collective focus on the importance of care and what it can make possible, enables persistence in the face of challenging circumstances.

### Ottawa Charter strategies in action

In this second theme, we analysed how strategies aligned to specific action areas of the Ottawa Charter were used by cat rescue organizations to create a health promoting setting for both humans and animals.

#### Reorienting the health system

Fostering programmes can be understood as a way of reorienting the health system for animals. This is a relatively recent move from earlier responses to caring for homeless animals, where animals were cared for in specialist shelters. Now, rescue organizations partner with community fosterers to care for animals at home, to provide a more health-promoting environment for animals.

Some rescue organizations were solely foster care based, while others maintained a central shelter supplemented by foster care. The choice to be entirely, or predominantly foster care based is in part resource driven but also reflects beliefs about what is best for animals.

We’ve discussed the options of ‘do you actually want to accumulate funds to be able to provide a shelter?’ And we’ve actually made the policy decision no, we don’t… we feel that actually fostering in foster homes is good, better for the cats and kittens in terms of as I say health wise and social wise. (ID 37)

Overall, the consensus was that a significant component of community-based foster care was the best choice for animal well-being, built on normal cat and human interactions in everyday settings.

We’re definitely moving towards an approach of getting all animals that can get out into foster. Just because we know that reduces the risk of deterioration in terms of behaviour, but also in terms of health. (ID24)

If they were all here, I wouldn’t have time to invest the attention to each individual cat that would be needed. It would slow the process down completely. Yeah, so for the cats wellbeing it’s good and also for the human wellbeing to be involved, to be a part of something, to be like they’re making a positive difference in the world. Yeah, it’s from both sides. Yeah. One can’t work without the other. (ID26)

These quotes highlight co-ordinators choosing foster care in the home, rather than an institutional setting, as a strategy to promote wellbeing for humans and animals, aligning with the Ottawa Charter emphasis on health being created in the settings of everyday life.

#### Strengthening community action

The Ottawa Charter action strategy of strengthening community action refers to the process of identifying and drawing on resources in the community to enhance social support. Several co-ordinators spoke of the importance of developing community networks to sustain their work. This included establishing partnerships with training organizations to access veterinary expertise through offering placements, alliances with businesses to host animals available for adoption, and working alongside community groups to both raise funds and promote fostering opportunities.

We get connections with people that then lead to connections with businesses…. We’ve got a great relationship with [organisation] which just opened and again for opening day they invited us to do the charity barbecue so it’s not… the outreach in the community doesn’t lead to us going, ‘Hey, can you help? Can you help?’ It’s like, ‘Hey, guys, guys we’ve got something, let’s help you guys.’ And then you get the social aspects of everybody connecting. (ID 26)

Some organizations had evolved these relationships over time. Others spoke of a turning point, where they realized an organization originally driven by the passion of a founder, needed to change to survive. The importance placed on wider community relationships aligns with the Ottawa Charter strategy of strengthening community action through drawing on community resources, building networks of mutual benefit and the benefits of sharing care across communities and institutions.

#### Creating supportive environments

While fostering first and foremost developed to create supportive environments for animals, co-ordinators also described intentions and actions to create supportive environments for fosterers and co-ordinators alike. Creating supportive environments for fosterers involved creating a sense of being part of a community.

And you know, the activity, fostering activity is not just about the cats, it’s also a social network, a fundraising network. It’s a bit of fun. … So it’s more than just feeding a cat. It’s becoming part of a community. That sounds very soft and sappy. But that’s what we’re aiming for, a larger, a larger community experience. (ID66)

a big piece we’re looking at in terms of retaining people. It’s making sure people know they’re part of this community, and we couldn’t do it without them. (ID 24)

The need for fosterers to feel part of a caring community is also demonstrated by the limitations of a service focused exclusively on the wellbeing of the animals.

We’ve had comments we’re not social enough. We’re not necessarily people focused…I just recently had a lady and she actually said to me, ‘You know, nobody ever asked me how I was’. And she’s right, we don’t do that. The question is, ‘How is the kitten?’. (ID 71)

Support through community building could create unexpected connections, as this co-ordinator speaking of the on-line face book page she set up, explained:

I think we have about 400 people on there now… And now we’ve got people, because we’ve been going since the 90s, we’ve got people who adopted, you know, over a decade ago who have joined the group and are showing photos of the animals that are now senior that were kittens when they were with [rescue organisation]. And we still have some of our foster homes available, like in our group from when those people adopted years and years ago. (ID 103)

Several co-ordinators also spoke of strategies they used to share work and responsibility as a way of caring for themselves. One noticeable pattern was moving from an initial model of a founder or one or two ‘going it alone’ to a model where organizational tasks were shared, reducing burnout and ‘spreading the load’. Others had intentionally established this wider network model of working, seeing it as better for the health of those involved.

we know our limits, we’ve got some funds in place, and we empower people to do what they want to do. … being foster based and moving from that one person crusade which is great for control to the governance group, you get more people involved, and you don’t burn out because when one of us burns out, the others wrap around, go it’s okay, this is your turn, you take a week off, we’ll cover that. (ID 66)

These quotes demonstrate different ways that co-ordinators work to create social connection, recognition and value, belonging and identity for human fosterers. These approaches at the organizational level align with the Ottawa Charter strategy of creating supportive environments to promote health.

#### Developing individual skills

Although traditional one-to-one skill development was provided through resources and training days, over half the rescues (63%) offered mentors for new fosterers. Twenty-three percent offered ‘foster buddies’. Both strategies allow for skill development through connection and relationships. Several co-ordinators mentioned their strategy of graduated exposure through matching. New fosterers would initially be matched with healthy confident animals. As fosterers gained experience they were moved on to fostering assignments that might prove to be more challenging. Matching was a process to develop individual skills through experience while paying attention to the impact of more challenging foster care relationships on fosterers.

So we might have first time fosterers, there’s no way we’re going to give them a wild feisty cat because that would scare them too much. So as their confidence and their knowledge grows, then they'll be able to take on [more]. (ID 26)

By providing different opportunities to develop skills and confidence in animal care, co-ordinators created conditions to develop fosterers’ capability, promoting wellbeing for both fosterers and animals.

## Discussion

Community fosterers live with, play, love, and care for animals in everyday settings. Through the lens of the Ottawa Charter, health promotion has care for others at its heart, and our study of cat fostering illustrates that care, supported by health promotion strategies.

Forty years ago, one of the contributions of the Ottawa Charter was to move beyond a focus on individual behaviour and recognize the importance of how the settings where we live, work, play and love affect health. Since the development of the Charter, much attention has focused on the importance of settings and wider environments for promoting health (Baum and Fisher 2014, Kokko and Baybutt 2022). Another Ottawa Charter claim—that care for ourselves and others creates health is an important but underexplored aspect of the charter. This is especially true in terms of our connections to nonhuman animals (Rock *et al*. 2015, Travers *et al*. 2022).

The example of rescue animal fostering highlights the complex nature of care for others, and the different forms that such care can take. These forms include care about, care for and the reciprocal or ‘give and take’ nature of care with others. It may require organizational persistence in the face of constraints in the wider environment. Co-ordinators’ perceptions of foster care relationships as both rewarding and challenging, mirror findings about the complex nature of permanent relationships with companion animals and their impact on human wellbeing (Applebaum *et al*. 2021, Toohey 2023). Specifically, our case study supports Toohey's concept of relational health promotion and her finding that valued relationships with animals can be both supportive and challenging. This fits within eudaimonic definitions of wellbeing that incorporate meaning and connection and allow for difficult experiences such as grief and loss. Our findings also affirm the value placed on contribution to others for well-being, even in the face of difficulty (Stephens *et al*. 2015).

Further, the study illustrates an application of the socioecological approach to health outlined in the Ottawa Charter. The socioecological approach stresses ‘*t*he need to encourage reciprocal maintenance and to take care of each other’ (World Health Organization 1986), but also the impact of the wider context on opportunities to care and contribute (Stephens *et al*. 2015, Beard *et al*. 2016). Our study reveals animal fostering work creating supportive relationships at the individual, organizational, and community level. Relationships between fosterers and cats in home environments are at the core of fostering, but co-ordinators also actively cultivate relationships among fosterers, between fosterers and the organization, and within the broader community to support their care work. Fosterers also connect and share stories through digital platforms, with or without direct organizational support, highlighting the increasing importance of digital communities as a form of connection for animal carers (Ngai 2023). While not identified as such by co-ordinators, relationship building is one of several strategies used in fostering that can be considered health promoting.

Ottawa Charter action strategies influence the broader care context. Such strategies are required for care giving organizations to be also considered health promoting (Dooris *et al*. 2022). The decision by animal rescue organizations to go beyond an institutional model to include everyday community-based care for animals represents one such strategy. Our study also found evidence of others. Co-ordinators strengthened community action by building alliances with community groups to recruit fosterers and fund foster care work, ensure veterinary support, offer training placements, and find homes for foster animals. They built supportive environments by connecting fosterers with each other and sharing responsibilities among foster programme staff. They developed individual skills through matching and mentoring, offering opportunities for fosterers to increase care responsibilities over time. These strategies show attention to providing the conditions for fostering to be health promoting, along with the opportunity for a caring connection with an animal, and to contribute to something meaningful.

Animal fostering not only aligns with the Ottawa Charter in the broad sense that care for others creates health, but through actions taken to create supportive environments. We can therefore confidently argue that rescue organization fostering programmes represent a version of health promotion in action.

### Implications for practice

Our study demonstrates that the Ottawa Charter provides a framework for recognizing animal fostering as health promoting. The Charter's clear applicability to this novel setting is a testament to its enduring value. However, this required extending the Charter's concept of ‘caring for others’ to encompass animals. This extension reveals a limitation in the original conceptualization of the Ottawa Charter—its anthropocentric framing.

More recent frameworks recognize the interdependence of humans and animals, aligning with indigenous perspectives that recognize the interconnectedness of living beings (Woodhouse *et al*. 2021, Stewart 2024, McPherson *et al*. 2026). Frameworks include One Welfare (Pinillos 2018), One Health (Stephen 2020) and ‘One Health Promotion’ approaches (Travers *et al*. 2022). Ecohealth and the planetary health perspective acknowledge connections between human health and ecological systems (Müller *et al*. 2025). What the Ottawa Charter offered that these broader frameworks do not, was a place for the individual, relational act of care as a health-promoting practice. It remains a missed opportunity—health promotion has yet to fully engage with human–animal relationships in everyday settings.

This gap is notable given the Ottawa Charter’s own principles of caring for others and addressing health equity. Our case demonstrates that when extended to include animals, the Ottawa Charter effectively reveals health-promoting work that would otherwise remain invisible. This suggests both the Charter’s enduring relevance and an opportunity for health promotion to build on recent calls to more explicitly address multispecies relationships, and consider the interests of other species as an equity issue (Mackenbach 2021).

Finally, the relational focus of the Ottawa charter extends to recognizing common ground with other sectors and working with them to promote health, aligning with health in all policies approaches (World Health Organization 2023). The use of fostering programmes represents a move by the animal rescue sector away from institutions to seeing responsibility for reducing animal homelessness as shared among individuals, community groups, and animal welfare professionals (Guenther and Hassen 2021). This mirrors the human health care sector's move to a greater focus on health promotion. It suggests the value of building alliances with the animal welfare sector to address the common determinants of human and animal homelessness, and prevent the unnecessary relinquishment of animals (McLennan *et al*. 2022, McDowall *et al*. 2024).

### Strengths and limitations

Our study is one of the first to apply an Ottawa charter lens to animal rescue settings. Its strengths include the initial systematic national survey of the Aotearoa-New Zealand animal rescue sector and its sequential design. Responses to the survey were used to ensure interview participants represented a diverse range of organizations from the initial group. The mixed method approach allowed us to capture both brief responses from 40 cat rescue organizations, and to explore issues in more depth with a smaller group of cat rescue foster programme co-ordinators. The study was limited to the perspectives of providers and organizers and does not represent the experiences of fosterers. While considering the wellbeing of the cats in fostering programmes to some extent, our Ottawa Charter lens focused on human perceptions of fostering.

## Conclusion

Despite its anthropocentric framing, the Ottawa Charter provides a valuable framework for health-promoting practice if ‘others’ are understood to include nonhuman animals. It also points towards the value of closer collaboration between the human and animal health sectors on issues important to both. Our case study illustrates how fostering embodies Ottawa Charter principles and makes the case for a broader definition of health promotion that includes human–animal relationships of care. Care for and about animals is central to animal fostering, and animal rescue organizations can be viewed as health-promoting settings employing strategies consistent with the action areas of the Ottawa Charter. Both individuals and environments are important for health, and our findings further highlight the challenging, yet rewarding and life-giving nature of care. Our study demonstrates the enduring contribution of the Ottawa Charter, while highlighting the need for evolution to maintain relevance in our multispecies world.

## Data Availability

The data underlying this article cannot be shared publicly due to the conditions of ethical approval that protect the privacy of individuals that participated in the study.

## Footnotes

1 We use the terms pet and owner occasionally in the paper, but pet and owner are increasingly being replaced by the terms companion animal and guardian or carer which reflects a ‘duty of care’ while ‘owner’ infers possession.
